# Contribution of potassium solubilizing bacteria in improved potassium assimilation and cytosolic K^+^/Na^+^ ratio in rice (*Oryza sativa* L.) under saline-sodic conditions

**DOI:** 10.3389/fmicb.2023.1196024

**Published:** 2023-08-28

**Authors:** Aniqa Nawaz, Zia Ul Qamar, Muhammad Usama Marghoob, Muhammad Imtiaz, Asma Imran, Fathia Mubeen

**Affiliations:** ^1^Microbial Physiology Laboratory, Soil and Environmental Biotechnology Division, National Institute for Biotechnology and Genetic Engineering College, Pakistan Institute of Engineering and Applied Sciences, Islamabad, Pakistan; ^2^Rice Lab, Plant Breeding and Genetics Division, Nuclear Institute of Agriculture and Biology College, Pakistan Institute of Engineering and Applied Sciences, Islamabad, Pakistan

**Keywords:** potassium solubilizing bacteria, salinity, cytosolic K^+^/Na^+^ ratio, assimilation of potassium through KSB, plant growth promoting salt-tolerant bacteria

## Abstract

Sodium-induced potassium (K^+^) deficiency is more prevalent in salt-affected soils. Plants experience K^+^ starvation thus cytosolic K^+^/Na^+^ ratio is lowered, which is a prerequisite for their survival. K^+^ enrichment in crops can be acquired *via* K-solubilizing bacteria as a sustainable green agriculture approach. This study was conducted to explore potent K-solubilizing bacteria from the rhizosphere of wheat, rice, and native flora grown in salt-affected soils in two distinct regions of Pakistan. The aim of this work was to evaluate the contribution of microbial consortiums to the improvement of K^+^ assimilation and cytosolic K^+^/Na^+^ ratios in rice crops under saline-sodic conditions. Among 250 bacterial isolates, 9 were selected based on their salt (11% NaCl) and alkali (9) tolerance and K-solubilization indices (1.57–5.67). These bacterial strains were characterized for their plant growth-promoting traits and identified based on 16S rRNA gene sequencing. A consortium of *five* strains, namely, *Enterobacter hormaechei, Citrobacter braakii, Pseudomonas putida, Erwinia iniecta*, and *Pantoea agglomerans*, was used as a bio-inoculant to evaluate its role in K^+^ assimilation, cytosolic K^+^/Na^+^ ratio, and subsequent yield enhancement in rice grown under saline-sodic conditions. The impact of applied consortium on rice was assessed under variable salt levels (Control, 40, 80, and 120 mM) in a pot experiment and under natural saline-sodic conditions in the field. Plant agronomical parameters were significantly higher in the bacterial consortium-treated plants, with a concomitant increase in K^+^-uptake in root and shoot (0.56 and 0.35 mg g^−1^ dry wt.) of the salt-tolerant rice variety Shaheen. The root K^+^/Na^+^ ratio was significantly improved (200% in 40 mM and 126% in 80 mM NaCl) and in the shoot (99% in 40 mM and 131% in 80 mM) too. A similar significant increase was also observed in the salt-susceptible variety Kainat. Moreover, grain yield (30.39 g/1,000 grains wt.) and biomass (8.75 g) of the rice variety Shaheen, grown in field conditions, were also improved. It can be concluded that K-solubilizing bacteria can be used as bio-inoculants, contributing to growth and yield increment *via* enhanced K-assimilation and cytosolic K^+^/Na^+^ ratio in rice crops under salt stress.

## Introduction

Pakistan is an agriculture-based country where agriculture contributes one-fifth of the total gross domestic product (GDP) (Usman, 2016). Rice (*Oryza sativa* L.) is an important agricultural commodity. It contributes 3% to value addition in agriculture and about 0.6% of GDP (Abdullah et al., 2015). It is the chief source of carbohydrates. It exhibits greater sensitivity to salinity among cereal crops (Carillo et al., 2011). Its grain yield starts to decline at 4 Ds/m and a 50% reduction is reported at 8 Ds/m soil salinity (Razzaq et al., 2020). The foremost inducement of soil salinization in arable lands is climate change and other related factors, including technological, industrial, and agricultural advancements and anthropogenic activities (Smajgl et al., 2015; Smajgl, 2018). A substantial decrease in crop production is caused by soil salinity, as it contributes significantly to the reduction of plant growth and yield (Hopmans et al., 2021; Li et al., 2022).

The presence of high concentrations of sodium (Na^+^) salts (soluble or insoluble) can induce nutrient deficiency, especially potassium (K^+^), in plants (Wakeel and Ishfaq, 2022a). Sodium stress imbalances the equanimity of K^+^ and Na^+^ concentration, which play a crucial role in plant growth; hence, the cytosolic K^+^/Na^+^ ratio is declined (Kumar et al., 2020). Potassium, a primary cationic macronutrient, is involved in a series of metabolic processes in plants, including the assimilation of CO_2_, improvement of nitrogen and phosphorus use efficiency (Dahuja et al., 2021), synthesis and translocation of sugars, and the osmotic adjustment under biotic and abiotic growth limiting factors through the regulation of more than 80% of enzymes (Wakeel and Ishfaq, 2022b). The prestige of K^+^ is analogous to nitrogen (N) for cereals (Ahanger et al., 2015). Sodium-induced potassium deficiency causes chlorosis in leaf margins, reduced biotic and abiotic stress resistance, and decreased qualitative and quantitative yield (Li et al., 2022). Severe deficiency can be observed at the beginning of intermodal elongation at the early reproductive stage, which aggravates with time (Zhu et al., 2020).

Although the soil is a huge reservoir of K^+^, 98% of its total contents exist in fixed form; however, only 2% is present in plant-available form (Jalali et al., 2021). Under saline-sodic conditions, Na^+^ competes with K^+^ for plant uptake and interferes with plant metabolism due to its chemical homology (Yan et al., 2021). The major causes of the aforesaid competition include either low K^+^ concentrations in the root zone or upregulation of nonselective cation channels (NSCCs) enforced by reactive oxygen species (ROS), which promote K^+^ leakage from the cell. Consequently, osmotic stress induced by Na^+^ influx causes plasma membrane depolarization, resulting in disruption of the cytosolic K/Na ratio (Wang et al., 2013). To sustain cellular membrane integrity, maintenance of a low Na^+^ concentration is mandatory, which is considered a key component in salt stress tolerance in plants (Wakeel, 2013). Optimum K^+^ concentration is crucial for adequate plant growth and can be achieved either by increasing K^+^ concentration in plant growth medium or by restricting Na^+^ influx through high-affinity potassium transporters (HKT) and NSCCs and the regulation of Na^+^/H^+^ antiporter systems on the plasma membrane and tonoplast (Zaman et al., 2015). These attempts will help to minimize competition between Na^+^ and K^+^ and permit the maintenance of cytosolic K^+^/Na^+^ in plants (Gul et al., 2019).

A number of strategies have been employed, mainly the use of inorganic and organic soil amendments (agrochemicals) for the eradication of high Na^+^ salt concentrations from the rhizosphere (Nawaz et al., 2020). However, these strategies are putting extra pressure on the country's economy (laborious crop management inputs) and causing unfriendly ecological impacts (reduced soil fertility, diversity of beneficial microbiota, and mineral nutrient depletion) (Mishra and Khare, 2021; Musa et al., 2022). Therefore, there is a dire need to adopt certain alternate sustainable, cost-effective, and eco-friendly measures to improve K^+^ concentration in saline-sodic soils (Wang et al., 2020). In this regard, rhizosphere engineering through the use of microbes serves as a fundamental constituent of phytoremediation technology (Deb et al., 2020). Plant growth-promoting rhizobacteria (PGPR) inhabiting stressful environments retain the potential to minimize stress through the release of primary and secondary metabolites (Egamberdieva et al., 2019). These osmolytes contribute to cellular osmotic adjustment, regulation of ionic transportation to sustain an optimal K^+^/Na^+^ ratio (a prerequisite for plant's salt stress tolerance) (Chen et al., 2020), and solubilization and mineralization of nutrients like phosphorus, potassium, and zinc (Alaylar et al., 2020; Daly et al., 2021). In addition, these microbes contribute to the fixation of atmospheric nitrogen through a wide range of extracellular and intracellular enzymes such as amylase, catalase, oxidase, cellulose, protease, ACC deaminase, iron chelating compounds (siderophores), and the production of exopolysaccharides (EPS) to assist in the formation of biofilm around the root to restrict Na^+^ ions intrusion *via* entrapment into biofilm (Bhise and Dandge, 2019).

Potassium-solubilizing bacteria (KSB) are those bacteria that improve the growth and productivity of plants grown under salt-affected conditions *via* the breakdown of complex K compounds into simpler ones, hence making them available to plants (Bahadur et al., 2019). These KSB weather the K^+^-bearing minerals *via* three mechanisms, primarily acidification, which breaks down the potassium aluminosilicate complex by releasing organic or inorganic acids into either potassium silicate or aluminum silicate in plant-available form (Bahadur et al., 2019). Second, the production of siderophores; organic acids form chelating compounds with Fe^+2^, Si^+4^, Ca^+2^, and Al^+3^ release K^+^ in the exchangeable K pool. Third, certain extracellular polymers, including proteins or EPS, are synthesized by microbes that make biofilm around the mineral rocks and weather them by a transformation of their morphology, hence liberating K^+^ into plant-available form (Jain et al., 2022). Various PGPR genera, such as *Bacillus, Acidothiobacillus, Paenibacillus, Pseudomonas, Enterobacter, Pantoea, Erwinia, Azospirillum, Marinococcus, Serratia, Streptomyces*, and *Azotobacter*, have been reported extensively to solubilize K and their subsequent contribution to improved plant growth and yield (Kour et al., 2020). Application of KSB is a sustainable measure to assimilate K^+^ and improve cytosolic K:Na in plants under K^+^ deficiency to restrict the use of chemical fertilizer (Sattar et al., 2019; Kour et al., 2020; You et al., 2020). Previous studies also illustrate the contribution of KSB in the growth and yield augmentation of major food (rice, wheat, maize, and sugarcane) and fiber (cotton) crops (Bahadur et al., 2019; Ali et al., 2021; Adnan et al., 2022; Soumare et al., 2022).

It was hypothesized that K-solubilizing bacteria contribute to the improvement of K assimilation and the cytosolic K^+^/Na^+^ ratio in rice under saline-sodic conditions. Therefore, the preliminary objective of this study was to isolate salt-tolerant K-solubilizing bacteria from salt-affected arable areas of Punjab and Khyber Pakhtunkhwa (KPK), Pakistan. Second, their subsequent role in salt stress suppression *via* improvement of K uptake and cytosolic K^+^/Na^+^ ratio in rice by increasing the available K pool. The output obtained from this study will be further employed to get better rice grain yields from cultivating salt-affected fields to address food security in the country.

## Materials and methods

### Collection of samples and analysis of soil physiochemical properties

Rhizospheric soil samples were collected from seven rice-cultivating districts, i.e., Faisalabad, Sheikhupura, Jhang, Hafizabad, Toba Tak Singh, Khanewal (Punjab), and native flora grown in salt-affected areas of district Kohat (KPK). The soil samples were randomly collected from each sampling site in triplicates at a depth of 6 cm from the surface, packed in zip-lock bags, brought into the lab, and stored at −4°C until further use. From each sample, 500 g of soil was used to analyze its physiochemical properties. Soil pH was determined by using a pH meter (Thomas, 1996), and electrical conductivity was found by using an EC meter (Rhoades, 1993). Available N and phosphorus (P) were found by following the Kjeldahl method (Yeomans and Bremner, 1991), the sodium bicarbonate method (Olsen, 1954), and the measurement of carbonates, bicarbonates, chloride, calcium, and magnesium were carried out through the calorimetry method. Quantification of Na^+^ and K^+^ was done by using a flame photometer (Simard, 1993), and organic matter and texture were determined by following the method described in Nelson and Sommers (1983) (Supplementary Table S1).

### Isolation of K-solubilizing bacteria

Rhizoshperic (soil tightly adhering to the roots) and root samples were taken to isolate K-solubilizing bacteria on Alexsandrow Agar media by following the protocol (Andrews, 2001). Colonies showing a halo zone were considered potent K-solubilizing isolates. Each distinct morphotype was subcultured to get single colonies. The pure cultures were stored at −20°C in a 20% glycerol solution. Endophytic bacterial strains were isolated by washing root samples thoroughly with sterilized distilled water, subsequently washing with 70% ethanol for 3 min, 0.1% H_2_O_2_ solution, and finally sterilized distilled water three times. Roots (1 g) were ground in a mortar and pestle under aseptic conditions and used for the isolation of endophytic bacteria through serial dilution and plating methods, as mentioned earlier.

### Quantitative estimation of K-solubilization, acid production, and salt-tolerance profiling of KSB

Salt tolerance of 250 distinct morphotypes was assessed by inoculating 1 ml of bacterial culture in 5 ml of LB broth supplemented with 3, 5, 7, 9 11, 13, and 15% sodium chloride (NaCl) concentrations and placing it under shaking conditions at 120 rpm for 3 days. Optical density (OD) was measured at 600 nm to enumerate bacterial growth spectrophotometrically (Andrews, 2001). Almost 63 bacterial isolates were tested to quantify the solubilized K in Alexsandrow Broth. For that purpose, overnight-grown bacterial cultures were transferred into 25 ml of Alexsandrow broth and placed in a shaking incubator at 28°C and 120 rpm for 7–10 days. The amount of solubilized K and pH (a measure to estimate acid production) of the filtrate were measured by using a flame photometer (Angraini et al., 2016) and a pH meter, respectively. Potassium chloride (KCl) was used to construct the standard curve.

### *In vitro* assessment of plant growth- promoting traits of selected KSB

Selected KSB were *in vitro* assessed for their plant growth-promoting traits, including solubilization of phosphorus (P) (Pikovskaya, 1948) and zinc (Zn) (Fal, 1996), production of siderophore (Schwyn and Neilands, 1987), exopolysaccharides (Gerhardt, 1994), and amylase (Peltier and Beckord, 1945), by following the standard protocols.

### Identification and phylogenetic analysis of KSB

Molecular identification of selected KSB was carried out through amplification of the 16S rRNA gene. Bacterial genomic DNA was extracted through the GeneGet Genomic DNA Purification Kit (Thermo Scientific) by following the manufacturer's protocol. Extracted DNA was quantified through a nanodrop spectrophotometer. Amplification of the 16S rRNA gene was conducted by using universal forward (PA 5′-AGACTTTGATCCTGCTCAG-3′) and reverse (PH 5′-AGGAGGTGATCCAGCCGCA-3′) primers and following the PCR profile reported by Ayyaz et al. (2016). The amplified PCR product was further cleaned through a gel extraction kit (Thermo Scientific) and submitted to Macrogen Inc., South Korea, for Sanger sequencing. Sequences were cleaned, assembled, and submitted to GenBank for accession numbers. Furthermore, phylogenetic analysis was performed using the Mega X software (Kearse et al., 2012).

### Compatibility and development of the KSB consortium

Cross-compatibility of five bacterial strains, namely, AN-K1 (AC # CP087390), AN-K22 (AC# OQ318272), AN-K49 (AC # OQ318273), AN-K75 (AC# 318276), and K86 (OQ318279), was checked by spot inoculation on Luria-Bertani (LB) agar plates. The co-occurrence of bacterial growth showed the compatibility of selected bacterial strains suitable to form a consortium. The consortium was developed by growing the bacterial strains in LB broth individually until the required bacterial population (10^−7^ to 10^−8^ CFU) was achieved. Then, all cultures were mixed together in a 1:1 ratio and further used to inoculate rice seeds and seedlings.

### Effect of the KSB consortium on germination and seedling vigor of rice

Seeds of the rice varieties Kainat and Shaheen were obtained from the Soil Salinity Research Institute, Pindi Bhattian, Hafizabad, Pakistan. Healthy seeds were surface sterilized with sodium hypochlorite solution (2%) for 4 min followed by successive washing five times with sterilized water. These seeds were soaked in KSB consortium for 2 h and placed on Petri plates containing a double layer of filter paper. The seeds were kept moist with 3 ml of sterilized water and placed in the dark until germination. After that, seeds were shifted to a growth room with a 12-h light and dark period at 28°C for 7 days. Data were recorded until a uniform germination rate was achieved for 3 consecutive days. After that, in order to evaluate the effect of KSB under variable salt concentrations (0, 40 mM, 80 mM, and 120 mM NaCl) on germination percentage (Islam et al., 2016), the seedling vigor index was measured using the formulas given below:


$$\begin{array}{l} \text{Germination percentage }(\text{GP})\text{ = Number of germinated seeds/}\\ \text{ Total number of seeds }\times \text{100}\\ \text{ Vigor index }(\text{V1})\text{ = Germination percentage/}\\ \text{ Plant height }\times \text{100} \end{array}$$

### *In planta* evaluation of the KSB consortium for growth, cytosolic K^+^/Na^+^ ratio improvement in rice, and soil exchangeable K^+^ pool

Rice varieties Kainat and Shaheen were used to study the contribution of the KSB consortium in the improvement of plant growth and the cytosolic K^+^/Na^+^ ratio (root and shoot), which is considered an essential character of salt tolerance (ST) in plants. Additionally, the contribution of the developed KSB consortium to the improvement of the soil exchangeable K^+^ pool was also assessed. Rice plant nurseries were sown at the end of May 2021. Rice seedlings were transplanted about 35 days after germination. At the time of transplantation, rice seedlings were dipped in a bacterial consortium for 1 h and then transferred to earthen pots containing 12 kg of homogenized soil. These pots were well watered in order to achieve moisture in the soil prior to rice transplantation. Four plants were established in pots for both varieties. The experiment was laid down based on a completely randomized design (CRD) with two identical sets comprised of four treatments (**T1** = 100% farmer-recommended fertilizer dose, **T2** = 20% reduced fertilizer dose, **T3** = 100% farmer-recommended fertilizer dose + Bacterial consortium, and **T4** = 20% reduced fertilizer dose + Bacterial consortium) and three replicates. After the establishment of rice seedlings, 20 days after transplantation, plants were classified into control plants and salt-stressed plants, and salt stress was applied. Tap water was used for the irrigation of control plants, while salt solutions containing 40 and 80 mM NaCl concentrations were used to irrigate stressed plants until the required concentration was achieved. Approximately 14 days after the imposition of salt stress, plant samples were collected to record the effect of salt stress on morphological and elemental status.

### Morphological parameters

Two plants/pots were collected and divided into shoot and root to record morphological parameters including root length, shoot length, and root and shoot fresh weight. For this purpose, the distance from the crown to the leaf tip was recorded as plant height, and the root length was measured up to the root tip. Root and shoot fresh weight was measured using an analytical balance.

### Evaluation of salt tolerance index in inoculated and un-inoculated plants

ST1 of each parameter was determined by following the formula:


$$\begin{array}{l} \text{Salt tolerance index }(\text{STI})\text{ = value observed under salt stress/}\\ \text{ value observed under normal}\\ \text{ conditions }\times \text{100} \end{array}$$

### Salt tolerance evaluation by membership fraction value (MFV) in inoculated and un-inoculated plants

A fuzzy comprehensive evaluation method using MFV (Chen et al., 2012) was adapted to evaluate the salt tolerance of two rice varieties under bacterially treated and non-treated conditions in the presence of variable levels of sodium chloride. The following equation was used to evaluate ST.


$$\begin{array}{l} {\text{X}}_{\text{i}}=\left(\text{X}-{\text{X}}_{min}\right)/\left({\text{X}}_{max}-{\text{X}}_{min}\right)\;\times 100 \end{array}$$

where X_i_ is the MFV of the salt tolerance index of a specific treatment, X is the actual measured value of a specific treatment, and X_*max*_ and X_*min*_ were the maximum and minimum values observed in all treatments (Ding et al., 2018). The average value of all MFVs of each treatment, ranging between 0 and 1, will be used to categorize the salt tolerance of rice varieties under inoculated and un-inoculated conditions. The higher the value of the mean MFV, the higher the tolerance of plants to the respective treatments.

### Determination of root and shoot K^+^, Na^+^, K^+^/Na^+^, and soil exchangeable potassium content

The dried shoot and root samples (1 g) were digested with sulfuric acid and hydrogen peroxide (Allen et al., 1985). The digested material was diluted up to 100 ml with distilled water. This diluent was further used to determine the concentration of K^+^ and Na^+^ by using a flame photometer in mg/L. Soil exchangeable potassium was estimated through digestion of a 1 g soil sample with the tri-acidic method (HNO_3_, H_2_SO_4_, and HClO_4_) in 9:5:1. The aforesaid procedure was adapted to dilute and enumerate the potassium content in digested soil samples by using a Flame photometer.

### Evaluation of KSB consortium for rice yield attributes and soil exchangeable K^+^ content under saline-sodic field conditions

A field experiment was conducted on farmland at SSRI, Pindi Bhattian, District Hafizabad, during the rice growing season in 2021. Seeds of two contrasting rice varieties, such as Shaheen and Kainat, were collected from SSRI for this experiment, and the experiment was designed in a randomized complete block design (RCBD) with three treatments: **T1** = 100% farmer-recommended fertilizer dose, **T2** = 20% reduced farmer-recommended fertilizer dose, and **T3** = 80% fertilizer + bacterial consortium in triplicates. At full maturity, the crop was harvested, and the yield-contributing components, including panicle length, number of fertile tillers, number of primary and secondary branches, total spikelets, fertility percentage, and 1,000 grains weight, were recorded. The soil samples were collected from each respective treatment and analyzed to estimate exchangeable potassium content through the tri-acid digestion method; the measurement of potassium was calculated through a flame photometer.

### Statistical analysis

All data from *in vitro* and *in planta* studies (pot and field experiments) were analyzed by ANOVA using the Statistix version 8.1 software. The least significant difference (LSD) was used to compare treatments at a confidence level of *p* = 0.05.

## Results

### Isolation of KSB

Rhizospheric soil samples from rice, wheat, and native flora were collected from eight districts in Pakistan. Out of a total of 250 morphotypes, 63 bacterial isolates were able to grow up to 11% NaCl concentration. While only nine isolates, namely, AN-K2, AN-K6, AN-K22, AN-K49, AN-K57, AN-K73, AN-K75, AN-K78, and AN-K86, were selected as potential K-solubilizers as they formed halo zones on Alexsandrow agar media plates after an incubation time of 7 days. Their solubilization indices ranged from 1.57 to 5.57, which exhibits their potential to solubilize inorganic K (Table 1). Among the nine selected isolates, AN-K49 showed the maximum solubilization index, followed by AN-K73, AN-K78, AN-K22, and AN-K75, respectively, while the least response was represented by AN-K2 and AN-K57.

**Table 1:**
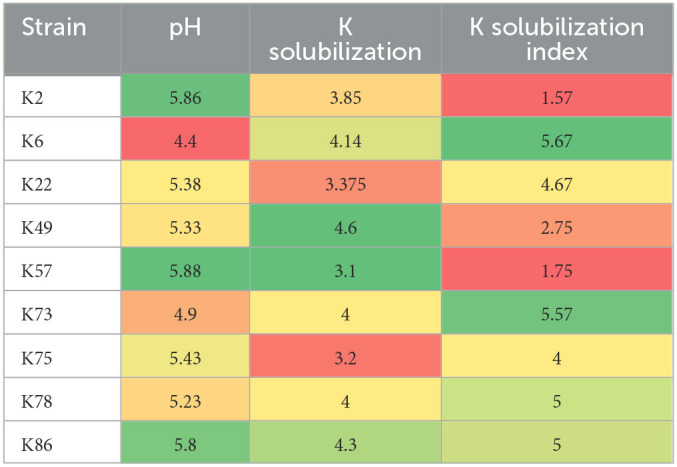
Heat map of KSB showing potassium solubilization index, Quantitative K released and subsequent pH reduction in Alexsandrow Broth media.

### Salt tolerance, acid production, and quantitative K-solubilization of selected KSB

All bacterial isolates were assessed for their salt tolerance on enriched media supplemented with 3, 5, 7, 9, 11, 13, and 15% NaCl concentrations through minimal inhibitory concentration. It was observed that all bacterial isolates showed remarkable growth up to 11% NaCl concentration, but none of them were found to grow at 11% NaCl. All selected bacterial strains solubilized potassium in Alexsandrow broth, with a subsequent decrease in the pH of the media. These bacterial strains solubilized potassium ranging from 4.6 to 2 mg/L with a decrease in pH of Alexsandrow broth up to 4.4. The maximum potassium content released in media after solubilization was observed by AN-K49, while the minimum amount of inorganic potassium was released by AN-K57 (3.1 mg/L), as shown in Table 1.

### Plant growth-promoting traits of KSB

All selected KSB strains showed their potential to solubilize inorganic phosphate on Pikovskaya agar plates containing tri-calcium phosphate as the sole insoluble phosphate source; PSI values ranged from 1.28 to 3.33, respectively. These bacterial isolates were also positive for zinc solubilization, and their ZSI ranged from 2.33 to 4.67. Moreover, all bacterial isolates were positive for siderophore production on CAS agar plates except AN-K22, while AN-K6, AN-K49, AN-K75, AN-K78, and AN-K86 showed average positive activity and the rest exhibited minor positive results. These bacterial isolates formed biofilm as an indication of their positive activity for exopolysaccharide production. The highest activity was observed in K86, while K6 and K57 showed minor results. On the other hand, the rest of the isolates showed minor positive EPS production, as illustrated in Table 2. Production of amylase enzyme on starch agar was observed among all selected bacterial isolates and showed clear areas around bacterial cultures after flooding the plates with Logol's solution after a 24-h incubation period.

**Table 2:**
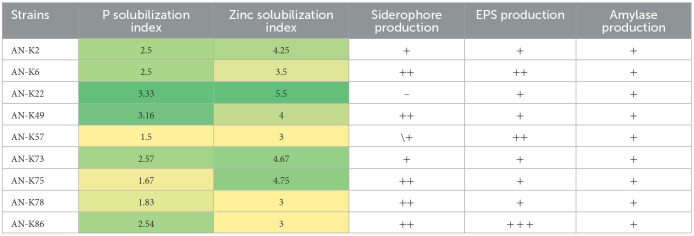
Plant growth promoting traits of selected KSB.

### Identification and phylogenetic analysis of KSB

**Figure 1:**
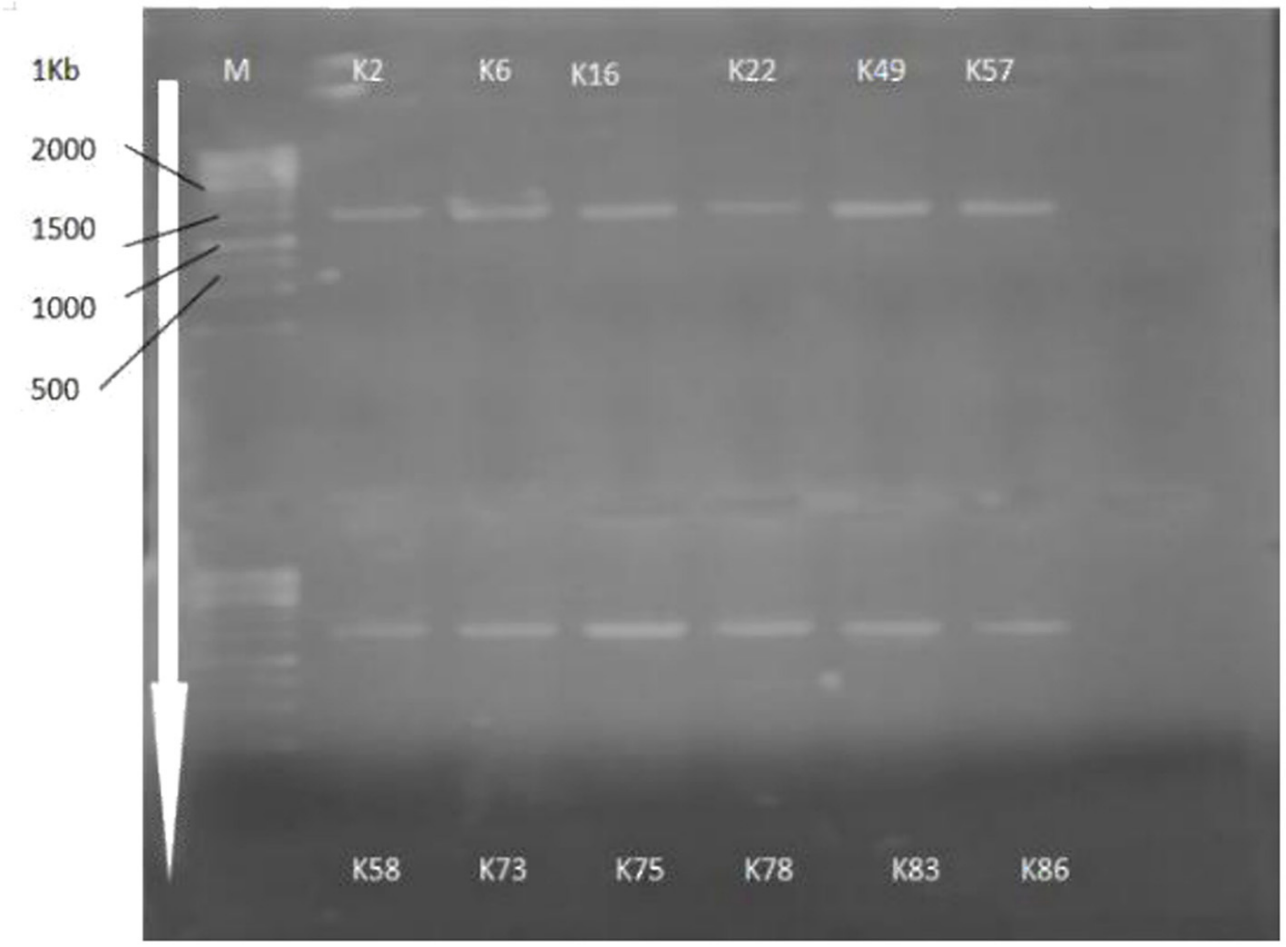
Amplification of 16S rRNA gene sequences of selected KSB strains.

**Figure 2:**
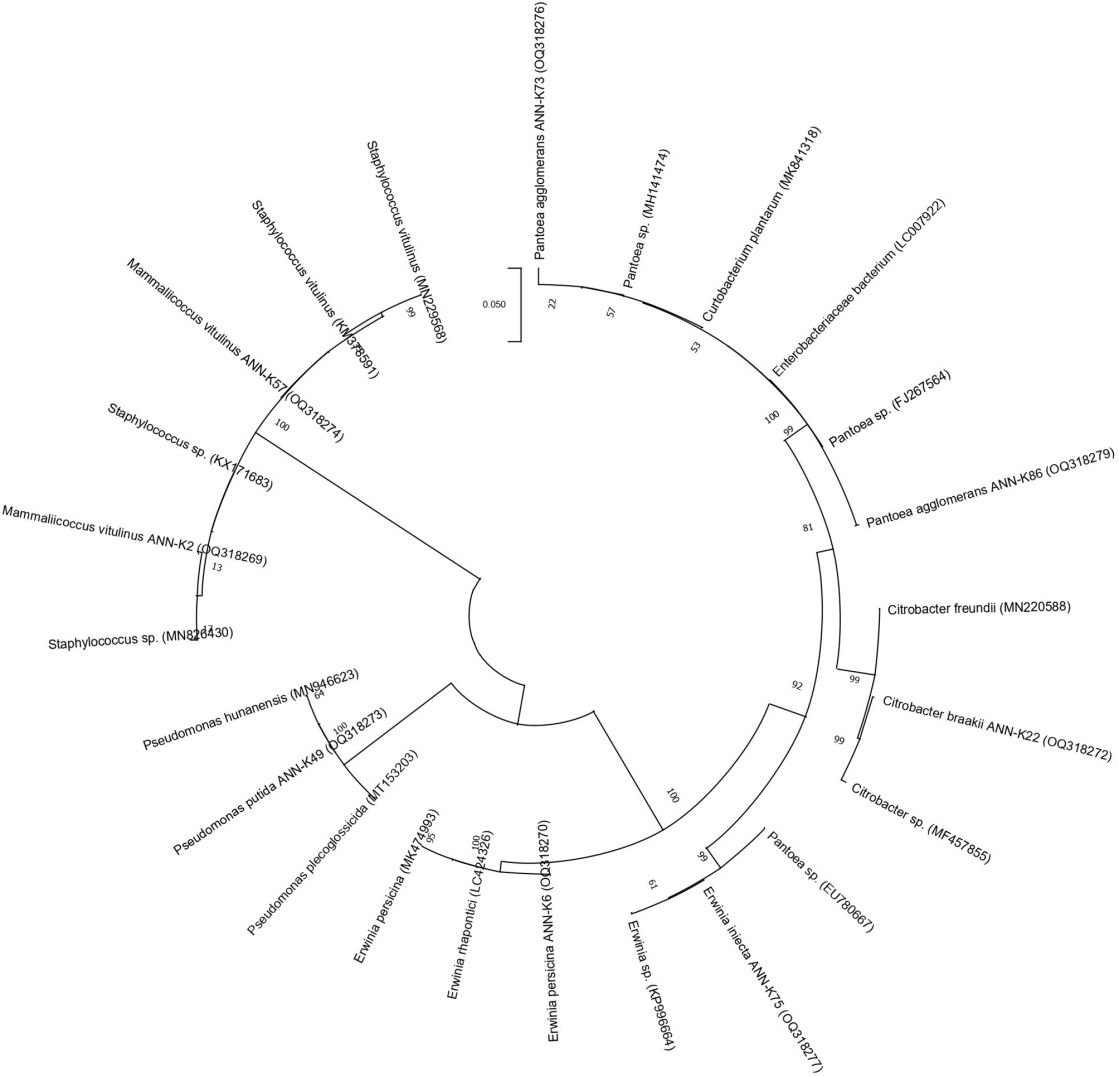
Dendrogram generated in Mega X software by selecting maximum likelihood similarity with 500 bootstrap method for sequenced KSB strains.

16s rRNA gene sequencing (Figure 1) using the Sanger sequencing platform was performed through Macrogen, Inc., South Korea. The sequencing outputs showed that these bacterial strains belonged to *Mammaliiococcus, Erwinia, Citrobacter, Pseudomonas*, and *Pantoea* spp. (Figure 2; Supplementary Table S2).

### Effect of the KSB consortium on germination and seedling vigor of rice

A plate bioassay was conducted to study the contribution of developed bacterial consortiums on germination percentage and seedling vigor index (VI) in contrasting rice varieties. Data depicted that the application of salinity did not affect the germination percentage in both varieties in inoculated plants even after the imposition of salt stress (4, 8, and 120 mM NaCl). However, seedling vigor in variety Shaheen was found to be decreased at 80 mM (51.05%) and 120 mM (20.23%) salt stress as compared with the inoculated control (VI = 850^a^) and those plants that received 40 mM (VI = 841^a^) salt stress. Maximum VI was observed in the plant that received the tap water. A decrease in seedling vigor was seen with the increased dose of NaCl in the plant's growth medium (soil), but this negative effect became less prominent in those plants that were treated with the bacterial consortium. In the salt-susceptible variety Kainat, a similar trend of response was observed. Whereas, inoculated control plants and those that were treated with 40 mM salt stress exhibited significantly higher SVI (738^a^ and 783^a^) as compared with 80 mM (655^b^) and 120 mM (616) salinities. Overall, the variety Shaheen performed much more efficiently in comparison to the salt-sensitive variety Kainat, as shown in Table 3.

**Table 3:** Seedling vigor index of ST-KSB inoculated rice varieties; Shaheen and Kainat, grown under variable levels of sodium chloride.

**Variety**	**Control**	**40 mM**	**80 mM**	**120 Mm**
Shaheen	850^a^	841^a^	416^c^	678^b^
Kainat	738^a^	783^a^	655^b^	616^b^

The mean values followed by different letters are showing significant difference at *p* > 0.05 according to LSD.

### *In planta* evaluation of KSB consortium for potassium, sodium, and potassium/sodium ratio improvement in rice and soil available potassium pool

#### Morphological characteristics

Application of the KSB consortium significantly increased the plant height among both rice varieties in T3 (consortium + 100% farmer recommended fertilizer dose) and T4 (consortium + 20% reduced fertilizer dose than farmer recommendation). However, a non-significant increase in shoot length was seen among consortium-treated plants. On the other hand, root length was significantly increased in inoculated (T3 and T4) plants in Shaheen, while in Kaint, only shoot length was found to be significantly improved in T3 and T4 in contrast with un-inoculated control plants. Root and shoot fresh weights were also observed to be improved by the implication of the PGPR consortium in both varieties.

#### Evaluation of salt tolerance

The MFV and the mean MFV of recorded traits were determined to evaluate the salt tolerance (ST) of inoculated plants as compared to un-inoculated plants. In a variety, Shaheen, un-inoculated plants (T1) treated with 40 mM NaCl represented the maximum ST, which was followed by T2, T3, and T4, respectively. Whereas, with the application of an increased dose of salt, *viz*., 80 mM NaCl, maximum ST was observed in plants treated with a 20% reduced fertilizer dose with bacterial inoculation (T4), followed by T1 (farmer-recommended fertilizer dose) and T3 (inoculated plants with 100% fertilizer dose). A minimum ST was observed in T2 (20% reduced fertilizer dose) plants (Table 4a).

**Table 4a:** Contribution of salt-tolerant KSB in improvement of rhizospheric soil potassium pool and morphological attributes of rice variety; **Shaheen**, grown under salt stress.

**Variety**	**Treatment**	**Plant length**	**Root length**	**Shoot length**	**Root FW**	**Shoot FW**	**No. of tillers**	**Soil K**
Shaheen	T1C	97.5^ab^	9^c^	85.25^b^	1.29^ab^	6.915^b^	4^b^	12.6^b^
	T2C	93^b^	15^a^	79^b^	0.6^c^	8.6^b^	6.5^a^	12.2^b^
	T3C	109.5^a^	13^b^	93.5^a^	1.51^a^	10.07^a^	5.5^a^	16.1^a^
	T4C	101^ab^	15.5^a^	96.5^a^	1.3^ab^	13.55^a^	6^a^	15.8^a^
	T1 40 Mm	89.5^c^	12.5^b^	78^bc^	0.85^c^	5.65^c^	5^a^	11^b^
	T2 40 Mm	93.5^b^	16^a^	77.5^bc^	0.795^c^	5.8^c^	4.5^b^	15.6^a^
	T3 40 Mm	107^a^	14^ab^	90.5^a^	1.45^a^	10.05^a^	5^a^	15^a^
	T4 40 Mm	95.5^b^	15.5^a^	82^b^	1.4^a^	10.95^a^	5^a^	10^bc^
	T1 80 Mm	78.33^d^	11^bc^	69.67^c^	1.2^ab^	4.9^c^	4^bc^	8^c^
	T2 80 Mm	84.33^c^	8.66^c^	67^c^	1.4^a^	4.13^c^	2.66^c^	7.7^c^
	T3 80 Mm	92.5^b^	13.5^b^	79^b^	1.2^ab^	7.95^b^	3.5^bc^	14.8^a^
	T4 80 Mm	111.5^a^	17.5^a^	86^b^	1.30^ab^	5.1^c^	4.5^b^	14^a^

Un-inoculated plants and 100% farmer recommended fertilizer dose **(T1)**, Un-inoculated plants and 20% reduced fertilizer dose than farmer recommendation **(T2)**, Inoculated plants and 100% farmer recommended fertilizer dose **(T3)**, Inoculated plants and 20% reduced fertilizer dose than farmer recommendation **(T4)**. Where plant, root and shoot length were measured in cm while fresh weight in g and exchangeable potassium in mg/kg^−1^. The mean values followed by different letters are showing significant difference at *p* > 0.05 according to LSD.

In the variety Kainat, the highest salt tolerance was observed in T4 plants, followed by T1, T3, and T2, when 40 mM NaCl stress was applied to un-inoculated and inoculated plants. On the other hand, when 80 mM NaCl was applied, T1 plants showed the highest ST (MFV value), followed by T4, T3, and T2, respectively. Overall, at 40 mM, T1-treated plants showed high tolerance, while the increase in salt concentration decreased their tolerance. A similar trend was also observed in T2 and T3 plants, but a quite different behavior was observed in T4, where high ST has been observed in the variety Shaheen. However, in the variety Kainat, salt tolerance was increased with bacterial inoculation in T3 and T4 in contrast with un-inoculated plants, i.e., T1 and T2. In the case of an increased dose of salt in the growth medium, bacterially inoculated plants with a 20% reduced fertilizer dose exhibited salt tolerance against un-inoculated plants (Table 4b).

**Table 4b:** Contribution of salt-tolerant KSB in improvement of rhizospheric soil potassium pool and morphological attributes of rice variety; **Kainat**, grown under salt stress.

**Variety**	**Treatment**	**Plant length**	**Root length**	**Shoot length**	**Root FW**	**Shoot FW**	**No. of tillers**	**Soil K**
Kainat	T1C	99.5^a^	18^a^	89^ab^	13.9^a^	24.7^a^	6^a^	12.5^b^
	T2C	91^ab^	19.5^a^	67^cd^	11.35^a^	26.3^a^	4^a^	12^b^
	T3C	97.5^a^	15^ab^	99.5^a^	4^b^	21.05^a^	3^b^	17^a^
	T4C	78^bc^	11.5^c^	95.5^a^	2.65^c^	8.3^c^	2^b^	16.2^a^
	T1 40 Mm	86^ab^	18^a^	71.5^c^	6.6^b^	19.75^ab^	5.5^a^	12^b^
	T2 40 Mm	85^b^	10.5^b^	64.5^d^	3.5^b^	15.25^b^	4^a^	11.7^b^
	T3 40 Mm	111.5^a^	12^b^	85.5^b^	1.65^c^	10.1^c^	4^a^	16.2^a^
	T4 40 Mm	107.5^a^	12.5^b^	71^c^	2.65^c^	15.8^b^	3.5^b^	15^a^
	T1 80 Mm	92.5^ab^	17^a^	83^b^	4.9^b^	18.1^ab^	5.5^a^	12^b^
	T2 80 Mm	87^ab^	13.5^b^	71^c^	3.5^b^	7.25^c^	5.5^a^	11.6^b^
	T3 80 Mm	85.5^b^	12^b^	70.5^c^	3.35^b^	8.05^c^	5^a^	15^a^
	T4 80 Mm	70.5^c^	11.5^c^	63.5^d^	2^c^	8.05^c^	4.5^a^	14^ab^

Un-inoculated plants and 100% farmer recommended fertilizer dose **(T1)**, Un-inoculated plants and 20% reduced fertilizer dose than farmer recommendation **(T2)**, Inoculated plants and 100% farmer recommended fertilizer dose **(T3)**, Inoculated plants and 20% reduced fertilizer dose than farmer recommendation **(T4)**. Where plant, root and shoot length were measured in cm while fresh weight in g and exchangeable potassium in mg/kg^−1^. The mean values followed by different letters are showing significant difference at *p* > 0.05 according to LSD.

#### Potassium/sodium index in root/shoot and soil potassium content

Dry root and shoot material was used to enumerate sodium and potassium contents. A further K+/Na+ ratio was calculated, a key trait for categorizing a plant's salt tolerance.

##### Root and shoot K^+^/Na^+^

Imposition of salt stress decreased the K^+^/Na^+^ ratio, which ranged from 110 to 70% in T1 and T2 (100 and 80% frt.) where plants were treated with 40 and 80 mM, respectively. Inoculated plants showed an increase in the K^+^/Na^+^ ratio as compared with un-inoculated and unstressed plants. So, it can be concluded that PGPR contributed to maintaining root K^+^/Na^+^ even under stressed conditions in the variety Shaheen. A similar trend of response was observed in a variety Kainat among inoculated and un-inoculated plants. Moreover, its value was significantly higher among inoculated salt-stressed plants in contrast with un-inoculated plants. Shoot K^+^/Na^+^ was also negatively affected upon exposure to salt stress, i.e., 40 and 80 mM. Inoculated plants exhibited a significant improvement in their value. A minor improvement was also observed in salt-treated plants in both rice varieties, Shaheen and Kainat (Tables 5a, 5b). Soil exchangeable potassium content was also significantly improved upon bacterial inoculation as compared with un-inoculated plants. Soil K content was also affected in the presence of 40 and 80 mM salt concentrations in the growth medium. However, the KSB consortium manifested a significant positive role in improving exchangeable K content in inoculated plants under stress conditions in the variety Shaheen. In the case of the variety Kainat, salt stress did not affect the exchangeable potassium pool. A significant increase in its content was observed in inoculated plants in normal and salt-stressed soil collected from plants' rhizospheric zones (Tables 5a, 5b).

**Table 5a:** Contribution of salt tolerant KSB in improvement of Sodium (Na^+^), Potassium (K^+^), and Potassium/Sodium ratio (K^+^/Na^+^) in rice variety, **Shaheen** (root and shoot), under normal and salt stressed conditions.

**Variety**	**Treatment**	**Root Na^+^**	**Shoot Na^+^**	**Root K^+^**	**Shoot K^+^**	**Root K^+^/Na^+^**	**Shoot K^+^/Na^+^**
Shaheen	T1C	23.33^cd^	169.567^c^	0.833^b^	3.3^b^	0.04^a^	0.019^b^
	T2C	28.13^cd^	137.567^d^	0.533^c^	2.633^bc^	0.018^c^	0.019^b^
	T3C	16.667^e^	65.933^f^	0.967^ab^	5.433^a^	0.05^a^	0.08^a^
	T4C	24.36^cd^	73.95^f^	1.267^a^	5.967^a^	0.05^a^	0.08^a^
	T1 40 mM	59.567^b^	181.183^b^	0.683^bc^	3^b^	0.011^c^	0.016^b^
	T2 40 mM	47.53^bc^	138.66^d^	0.433^c^	2.067^bc^	0.009^c^	0.014^b^
	T3 40 mM	52.56^bc^	126.48^de^	0.67^bc^	3.5^b^	0.03^b^	0.027^ab^
	T4 40 mM	35.667^c^	115.5^de^	0.683^bc^	2.167^c^	0.019^c^	0.018^b^
	T1 80 mM	36.383^c^	198.667^b^	0.5^c^	2.833^bc^	0.015^c^	0.014^b^
	T2 80 mM	76.767^a^	420.3^a^	0.417^c^	1.05^c^	0.005^d^	0.002^c^
	T3 80 mM	48.76^bc^	167.033^c^	0.64^bc^	2.833^bc^	0.013^c^	0.016^b^
	T4 80 mM	40.567^c^	195.35^b^	0.63^bc^	1.85^c^	0.015^c^	0.009^d^

Un-inoculated plants and 100% farmer recommended fertilizer dose **(T1)**, Un-inoculated plants and 20% reduced fertilizer dose than farmer recommendation **(T2)**, Inoculated plants and 100% farmer recommended fertilizer dose **(T3)**, Inoculated plants and 20% reduced fertilizer dose than farmer recommendation **(T4)**. Where root/shoot sodium and potassium contents were measured in mg g^−1^ dry wt. The mean values followed by different letters are showing significant difference at *p* > 0.05 according to LSD.

**Table 5b:** Contribution of salt tolerant KSB in improvement of Sodium (Na^+^), Potassium (K^+^), and Potassium/Sodium ratio (K^+^/Na^+^) in rice variety, **Kainat** (root and shoot), under normal and salt stressed conditions.

**Variety**	**Treatment**	**Root Na^+^**	**Shoot Na^+^**	**Root K^+^**	**Shoot K^+^**	**Root K^+^/Na^+^**	**Shoot K^+^/Na^+^**
Kainat	T1C	30.55^b^	79.03^d^	0.9^b^	3.53^a^	0.029^ab^	0.04^a^
	T2C	29.67^b^	81.683^d^	1.467^a^	3.43^a^	0.047^a^	0.04^a^
	T3C	28.28^b^	60.67^e^	1.56^a^	3.71^a^	0.05^a^	0.06^a^
	T4C	25.46^bc^	58.95^ef^	1.38^ab^	3.51^a^	0.05^a^	0.05^a^
	T1 40 mM	48.6^a^	133.283^c^	0.86^b^	3.46^a^	0.017^c^	0.026^b^
	T2 40 mM	30.36^b^	87.23^ab^	0.63^d^	2.85^b^	0.02^b^	0.03^ab^
	T3 40 mM	30.48^b^	126.1^c^	0.95^b^	3.86^a^	0.03^ab^	0.03^ab^
	T4 40 mM	29.01^b^	66.5^e^	0.883^b^	3.18^ab^	0.03^ab^	0.04^a^
	T1 80 mM	49.03^a^	315.5^a^	0.69^d^	2.89^b^	0.01^c^	0.009^b^
	T2 80 mM	41.43^a^	188.2^b^	0.35^e^	2.63^b^	0.008^c^	0.013^b^
	T3 80 mM	39.86^a^	171.6^b^	0.83^bc^	2.96^b^	0.02^ab^	0.017^b^
	T4 80 mM	38.68^a^	76.1^d^	0.783^c^	2.76^b^	0.02^ab^	0.03^ab^

Un-inoculated plants and 100% farmer-recommended fertilizer dose **(T1)**, Un-inoculated plants and 20% reduced fertilizer dose than farmer recommendation **(T2)**, Inoculated plants and 100% farmer-recommended fertilizer dose **(T3)**, Inoculated plants and 20% reduced fertilizer dose than farmer recommendation **(T4)**. Where root/shoot sodium and potassium contents were measured in mg g^−1^ dry wt. The mean values followed by different letters are showing significant difference at *p* > 0.05 according to LSD.

#### Effect of KSB on yield parameters of rice and soil potassium status

**Figure 3:**
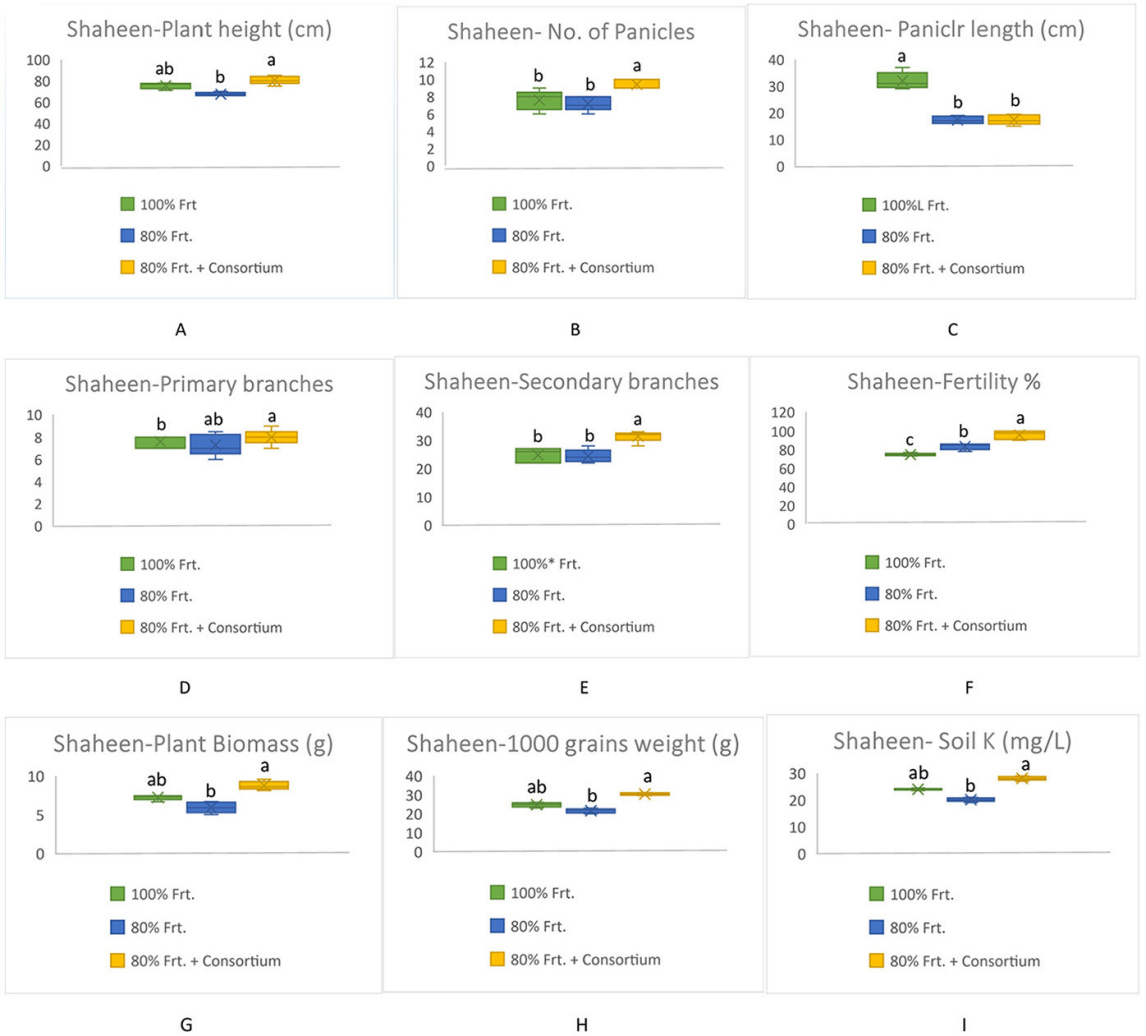
Contribution of KSB in the improvement of grains yield and its related components in rice variety, Shaheen and soil exchangable potassium content under saline-sodic conditions **(A)** Plant height (cm), **(B)** No. of Panicles, **(C)** Panicle length (cm), **(D)** Primary branches, **(E)** Secondary branches, **(F)** Fertility %, **(G)** Plant biomass (g), **(H)** 1000 grains weight (g), Soil K^+^ (mg/kg). The mean values followed by different letters are showing significant difference at *p* > 0.05 according to LSD.

**Figure 4:**
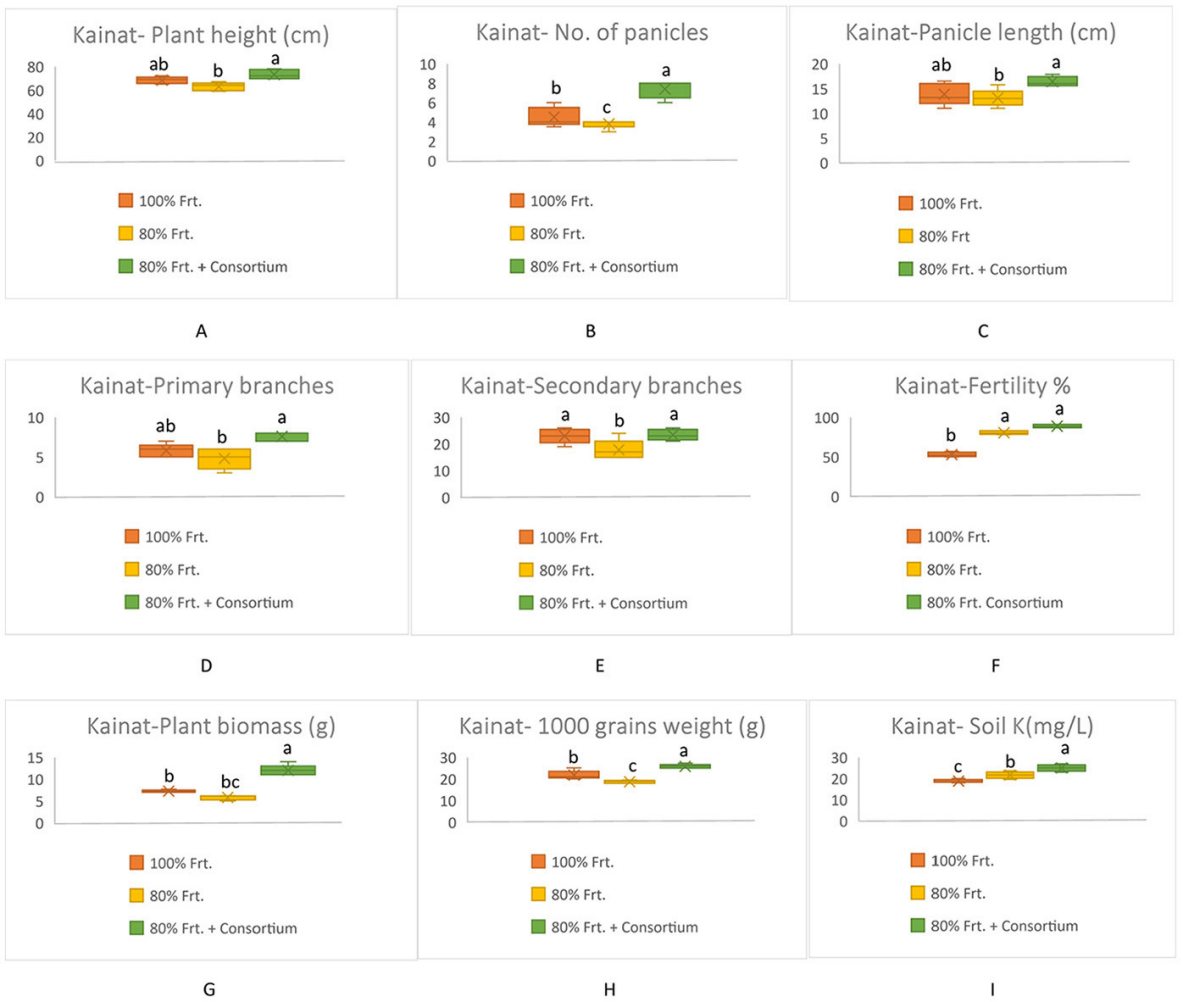
Contribution of KSB in the improvement of grains yield and its related components in rice variety, Kainat and soil exchangable potassium content under saline-sodic conditions **(A)** Plant height (cm), **(B)** No. of Panicles, **(C)** Panicle length (cm), **(D)** Primary branches, **(E)** Secondary branches, **(F)** Fertility %, **(G)** Plant biomass (g), **(H)** 1000 grains weight (g), Soil K^+^ (mg/kg). The mean values followed by different letters are showing significant difference at *p* > 0.05 according to LSD.

All yield-related components were significantly increased among KSB consortium-treated plants in both varieties; however, Shaheen presented much better performance as compared to Kainat when grown under naturally saline-sodic soil conditions. A similar response was observed in soil exchangeable potassium content, which was determined by collecting soil from treated and untreated plots. Overall, it can be concluded that inoculation by the KSB consortium improved the soil exchangeable potassium content in the rhizospheric zone, which ultimately contributed to rice yield augmentation in salt-affected soil conditions (Figures 3, 4).

## Discussion

Global food security and agricultural productivity are directly affected by climate change and its subsequent sequel of soil salinity (Subiramani et al., 2020). Yield penalties of up to 20–50% have been recorded among major agricultural commodities, including wheat, rice, and maize (Egamberdieva et al., 2019). So, it is mandatory to adopt an integrated approach to counter such an alarming situation. The Na^+^-induced K^+^ deficiency in salt-affected arable lands hinders plant growth and development because K^+^ serves as key macronutrients in metabolism and development, i.e., photosynthesis, translocation of sugars from source to sink, activation of enzymes, sugars and ATP production, and abiotic and biotic stress resilience (Wolde, 2016). Potassium is the second most important nutrient after N that has a direct correlation with growth promotion (Meena et al., 2014).

The exploitation of KSB, a green biotechnology approach, can be a sustainable substitute for agrochemicals (Das and Pradhan, 2016) that mobilize unavailable nutrients into a soluble form by releasing certain organic or inorganic acids, chelating compounds, or EPS (Meena et al., 2016). This study was designed to explore KSB in salt-affected arable lands of Punjab and KPK provinces in Pakistan, with the potential to survive in saline-sodic soils, and their subsequent role in yield augmentation of paddy crops *via* aggrandizing K^+^ pool in the root zone and subsequent maintenance of the plant's cytosolic K^+^/Na^+^ ratio under salt stress conditions. Previous studies explained the contribution of KSB to surging crop growth and yield (Ashfaq et al., 2020; Beaicknejad Daroonkolaei et al., 2023), but the exploration of KSB in salt-affected agricultural soils in rice cultivating areas in Punjab and microbiota residing in salt-affected areas of Kohat, KPK, Pakistan, is still unexplored.

Exploration of KSB in salt-affected soils enabled us to screen nine KSB belonging to genera, *Pseudomonas, Enterobacter, Citrobacter, Erwinia, Mammaliiococcus*, and *Pantoea* sp., respectively. These bacteria were viable up to 11% NaCl salt in growth medium and solubilized potassium ranged from 2 to 4.6 mg/L K+ with the subsequent decrease (4.4–6.09) in pH, which was initially adjusted at 7 in Aexsandrow broth media. These outcomes suggest that KSB was capable of producing certain acids, either organic or inorganic, which contributed to the breakdown of the potassium aluminum silicate complex and released inorganic K, hence increasing the K content (Meena et al., 2016). Olaniyan et al. (2022) described previously that K^+^ secrete organic acids, such as tartaric acid, citric acid, and oxalic acid, to solubilize the mineral K^+^ and make it accessible to plants.

We further evaluated these salt-tolerant KSBs for various plant growth-promoting assays *in vitro*. All selected isolates were capable of solubilizing inorganic tricalcium phosphate in Pikovskaya agar (PSI = 3.33–1.28) and zinc oxide (ZSI = 5.5–2.33) in tris-minimal media, respectively. Moreover, these isolates were found to be positive for their potential to produce exopolysaccharides, siderophores, and amylase activity. Only *Citrobacter* did not produce a change in coloration on CAS agar plates, exhibiting their negative response to siderophore production. The capability of acid, EPS, and siderophore production in selected KSB supports the previously reported studies that indicate that mineral-solubilizing bacteria adapt any of the aforesaid mechanisms for making minerals available to plants (Kour et al., 2020).

We evaluated the selected KSB consortium, comprising five bacterial strains, on germination percentage and seedling vigor index in the presence of varying NaCl concentrations (0, 40 mM, 80 mM, and 120 mM). Almost 100% germination was observed in all inoculated, un-inoculated stressed, and unstressed plants. However, the seedling vigor index declined upon exposure to salt stress at 80 and 120 mM. This negative effect of salt stress was less prominent in inoculated plants as compared with un-inoculated plants. Ferraz Helene et al. (2022) reported that the P and K solubilizer *Enterobacter* spp. increased seedling vigor, germination %, and P and K uptake by okra plants under salt stress. Jana et al. (2023) demonstrated the positive contribution of *Citrobacter, Pantoae*, and *Flavobacterium* in improving seedling vigor and germination rate. In terms of improved tomato plant growth, tissue K^+^ content, and soil residual matter (Raji and Thangavelu, 2021), similar outcomes of K-solubilizing *Bacillus* and *Bruckholdoria* sp. were reported. Naing et al. (2021) reported that P and K-solubilizing and N_2_ fixer *Erwinia precinct* and *Pseudomonas* contribute positively to improving chili plant growth parameters, including shoot biomass, shoot, and root ratio, as well as biochemical stress markers including chlorophyll, flavonoid's, and sugar content. Bahadur et al. (2019) proposed the conclusion of their study; salt-tolerant ACC deaminase-producing PGPR, including *Bacillus* sp. and *Pseudomonas putida*, improve wheat growth by modulating its morpho-physiological and biochemical attributes under saline conditions.

The microbial consortium improved the plant's traits (root/shoot K^+^/Na^+^ ratio and soil exchangeable K^+^) under stressed and normal conditions in both rice varieties. So, it can be claimed that the KSB consortium might be the best option to achieve sustainable development goals by minimizing the input of agrochemicals. Bahadur et al. (2019) reported similar findings that KSB can be used as an eco-friendly substitute for chemical potash applications, which has posed both human and environmental health concerns due to its excessive usage. The field application and assessment of the KSB consortium demonstrated a significant yield gain in the Shaheen variety in comparison to both the Kainat variety and the control treatment. This variant response of two rice varieties can be regarded as the potential of inoculated KSB varying with the plant's genotype, exudates, developmental stage, and compatibility with growth conditions. Yaghoubi Khanghahi et al. (2019) confirmed that KSB when studied for their growth promotion in rice in semi-controlled (pots) and natural (field) conditions, the inoculated plants showed significantly increased grain yield and dry biomass as compared with un-inoculated plants.

This study allowed us to explore the potential KSB plant growth stimulating rhizospheric bacteria residing in salt-affected agricultural areas of Punjab and KPK provinces of Pakistan. The developed microbial K-solubilizing consortium can be considered a sustainable, eco-friendly, and cost-effective alternative to agrochemicals. From this study, we can conclude that KSB acquires various mechanisms, either in a combination of two or three, to mobilize fixed K and subsequently increase the K-pool, which in turn improves available K-content for plants grown under saline-sodic or K-deficient conditions. Hence, it improves the plant's K^+^/Na^+^ cytosolic ratio, which ultimately increases crop productivity, both qualitatively and quantitatively, through the induction of salt stress tolerance.

## Conclusion

Offshoots of the proposed study deduced that rice cultivation in salt-affected lands in Punjab and KPK provinces of Pakistan comprises a rich population of salt-tolerant KSB, which can be employed as a sustainable tool to minimize grain yield penalties caused by salt stress through the improvement of the morphological and mineral nutrition status of rice crops. These KSB improved the K-pool in the rhizospheric zone of inoculated plants, hence improving growth by maintaining the cytosolic K+/Na+ ratio in plant tissues and enhancing potassium acquisition. Further studies based on the molecular basis of the key mechanisms adapted by KSB strains to solubilize fixed K and multi-locational application can lead to the development of an effective potash biofertilizer to fortify K-stressed (normal or salt-affected) soils in the country.

## Data availability statement

The datasets presented in this study can be found in online repositories. The names of the repository/repositories and accession number(s) can be found in the article/Supplementary material.

## Author contributions

AN executed, analyzed, and prepared the manuscript. FM and ZQ designed, supervised, and reviewed the manuscript. MM, MI, and AI reviewed the manuscript and helped in data analysis. All authors contributed to the article and approved the submitted version.
