# Supplementary material for: Contribution of potassium solubilizing bacteria in improved potassium assimilation and cytosolic K+/Na+ ratio in rice (Oryza sativa L.) under saline-sodic conditions
Source: Front Microbiol. 2023 Aug 28;14:1196024. doi: 10.3389/fmicb.2023.1196024 (PMC10497963; doi:10.3389/fmicb.2023.1196024)
Supplement: Supplementary file 1 [file Data_Sheet_1.pdf]

Supplementary S1 | Physico-chemical properties of rice cultivating major districts of Punjab and salt affected area of Khyber Pakhtunkhwa

Physico-chemical attributes of soil from district Faisalabad

Sr. No.	Parameter	Values	Units
1	**EC_e**	10.73	dS/m
2	**pH**	8.41	
3	**Sodium**	562	mg/L
4	**Potassium**	2.44	mg/L
5	**Calcium**	240	mg/L
6	**Magnesium**	51	mg/L
7	**Chloride**	999.69	mg/L
8	**Available Nitrogen**	1.4	mg/L
9	**Available Phosphorus**	19.6	mg/Kg
10	**Carbonates**	NIL	mg/L
11	**Bicarbonates**	561.2	mg/L
12	**Texture**	Clay loam	
13	**Organic matter**	1.39	%

Physico-chemical attributes of soil from district Sheikhupura

Sr. No.	Parameter	Values	Units
1	**EC_e**	7.42	dS/m
2	**pH**	8.59	
3	**Sodium**	549	mg/L
4	**Potassium**	2.4	mg/L
5	**Calcium**	240	mg/L
6	**Magnesium**	60	mg/L
7	**Chloride**	872.72	mg/L
8	**Available Nitrogen**	6.3	mg/L
9	**Available Phosphorus**	11.2	mg/Kg
10	**Carbonates**	NIL	mg/L
11	**Bicarbonates**	122	mg/L
12	**Texture**	Clay loam	
13	**Organic matter**	1.56	%

Physico-chemical attributes of soil from district Hafizabad

Sr. No.	Parameter	Values	Units
1	**EC_e**	2.29	dS/m
2	**Ph**	8.24	

3	**Sodium**	823	mg/L
4	**Potassium**	2.4	mg/L
5	**Calcium**	240	mg/L
6	**Magnesium**	420	mg/L
7	**Chloride**	999.69	mg/L
8	**Available Nitrogen**	7	mg/L
9	**Available Phosphorus**	45.8	mg/Kg
10	**Carbonates**	NIL	mg/L
11	**Bicarbonates**	538.8	mg/L
12	**Texture**	Sandy clay loam	
13	**Organic matter**	0.26	%

Physico-chemical attributes of soil from district Jhang

Sr. No.	Parameter	Values	Units
1	**EC_e**	7.98	dS/m
2	**pH**	8.59	
3	**Sodium**	851	mg/L
4	**Potassium**	1.99	mg/L
5	**Calcium**	1160	mg/L
6	**Magnesium**	360	mg/L
7	**Chloride**	2124.34	mg/L
8	**Available Nitrogen**	4.9	mg/L
9	**Available Phosphorus**	13.4	mg/Kg
10	**Carbonates**	NIL	mg/L
11	**Bicarbonates**	854	mg/L
12	**Texture**	Sandy loam	
13	**Organic matter**	1.16	%

Physico-chemical attributes of soil from district Toba Tak Singh

Sr. No.	Parameter	Values	Units
1	**EC_e**	5.26	dS/m
2	**pH**	7.91	
3	**Sodium**	823	mg/L7.91
4	**Potassium**	1.98	mg/L
5	**Calcium**	900	mg/L
6	**Magnesium**	20	mg/L
7	**Chloride**	1124.65	mg/L
8	**Available Nitrogen**	6.3	mg/L

9	**Available Phosphorus**	10.0	mg/Kg
10	**Carbonates**	NIL	mg/L
11	**Bicarbonates**	56.12	mg/L
12	**Texture**	Sandy loam	
13	**Organic matter**	0.86	%

Physico-chemical attributes of soil from district Khanewal

Sr. No.	Parameter	Values	Units
1	**EC_e**	8.16	dS/m
2	**pH**	8.99	
3	**Sodium**	931	mg/L
4	**Potassium**	1.79	mg/L
5	**Calcium**	100	mg/L
6	**Magnesium**	220	mg/L
7	**Chloride**	999.69	mg/L
8	**Available Nitrogen**	2.1	mg/L
9	**Available Phosphorus**	13.0	mg/Kg
10	**Carbonates**	2400	mg/L
11	**Bicarbonates**	1220	mg/L
12	**Texture**	Clay loam	
13	**Organic matter**	0.98	%

Physico-chemical attributes of soil from district Kohat

Sr. No.	Parameter	Values	Units
1	**EC_e**	39.9	dS/m
2	**pH**	7.86	
3	**Sodium**	1011	mg/L
4	**Potassium**	2.9	mg/L
5	**Calcium**	3040	mg/L
6	**Magnesium**	680	mg/L
7	**Chloride**	32789.92	mg/L
8	**Available Nitrogen**	2.1	mg/L
9	**Available Phosphorus**	2.0	mg/Kg
10	**Carbonates**	NIL	mg/L
11	**Bicarbonates**	48.8	mg/L
12	**Texture**	Calcareous loam	

13	**Organic matter**	0.32	%

Supplementary S2 | Sequencing and BLAST results

Code	Name	Query Cover	E value	Percent identity	Acc. Len
**ANN-K2**	*Mammaliicoccus vitulinus*	100%	0.0	99.46%	**OQ318269**
**ANN-K6**	*Erwinia persicina*	100%	0.0	99.84%	OQ318270
**ANN-K22**	*Citrobacter braakii*	100%	0.0	99.92%	OQ318272
**ANN-K49**	*Pseudomonas putida*	100%	0.0	99.92%	OQ318273
**ANN-K57**	*Staphylococcus vitulinus*	100%	0.0	99.92%	OQ318274
**ANN-K73**	*Pantoea agglomerans*	100%	0.0	98.78%	OQ318276
**ANN-K75**	*Erwinia iniecta*	100%	0.0	99.85%	OQ318277
**ANN-K86**	*Pantoea agglomerans*	100%	0.0	99.69%	OQ318279
